# From Species to Regional and Local Specialization of Intestinal Macrophages

**DOI:** 10.3389/fcell.2020.624213

**Published:** 2021-02-18

**Authors:** Cynthia Arroyo Portilla, Julie Tomas, Jean-Pierre Gorvel, Hugues Lelouard

**Affiliations:** ^1^Aix Marseille Univ, CNRS, INSERM, CIML, Marseille, France; ^2^Departamento de Análisis Clínicos, Facultad de Microbiología, Universidad de Costa Rica, San José, Costa Rica

**Keywords:** intestinal immunity, macrophages, microbiota, phagocytosis, stromal microenvironment, dietary antigens, metabolites, antigen sampling

## Abstract

Initially intended for nutrient uptake, phagocytosis represents a central mechanism of debris removal and host defense against invading pathogens through the entire animal kingdom. In vertebrates and also many invertebrates, macrophages (MFs) and MF-like cells (e.g., coelomocytes and hemocytes) are professional phagocytic cells that seed tissues to maintain homeostasis through pathogen killing, efferocytosis and tissue shaping, repair, and remodeling. Some MF functions are common to all species and tissues, whereas others are specific to their homing tissue. Indeed, shaped by their microenvironment, MFs become adapted to perform particular functions, highlighting their great plasticity and giving rise to high population diversity. Interestingly, the gut displays several anatomic and functional compartments with large pools of strikingly diversified MF populations. This review focuses on recent advances on intestinal MFs in several species, which have allowed to infer their specificity and functions.

## Introduction

The innate immune system encompasses different defense mechanisms selected over evolutionary time and encoded in the germline, hence passed to offspring with only minor refinements. Genome sequencing has established that much of these defense systems are conserved across animal phyla, reflecting their remarkable effectiveness and versatility (Litman and Cooper, 2007). These conserved defense mechanisms include the complement system, pattern recognition receptors (PRRs), and phagocytosis. The complement system is an ancient component of immunity that likely evolved from protection of the unicellular protists to essential defense functions in the blood of vertebrates (Elvington et al., 2016). Classical PRRs, such as Toll-like receptors (TLRs), C-type lectins, NOD-like receptors (NLRs), and perforin-2/MPEG-1, are already identified in non-bilaterian animals (Traylor-Knowles et al., 2019). Phagocytosis, from ancient Greek meaning “cell eating,” is typically an eukaryote-specific process that consists in the ingestion of particulate matters larger than 0.4 μm by a cell through invagination of its membrane (Mills, 2020). Inside the Eukaryota domain, plant cells are not able to phagocyte due to their rigid cell wall. In addition, no phagocytosis has been reported in fungi, with the exception of the parasitic fungus *Rozella allomycis* (Yutin et al., 2009). By contrast, protists use phagocytosis for the intake of nutrients from the environment where these unicellular organisms reside. In parasitic infections, such as trichomoniasis, the protozoan *Trichomonas vaginalis* uses phagocytosis to ingest *Saccharomyces cerevisiae* cells, vaginal epithelial cells, leucocytes, and erythrocytes (Pereira-Neves and Benchimol, 2007). Phagocytosis involves cell membrane receptors for target recognition. Thus, the scavenger receptor cysteine-rich (SRCR) domain family of receptors is encoded in the genomes from the most primitive sponges to mammals (Dzik, 2010). Receptors for phagocytosis bind the particles either directly or via opsonins (antibodies or complement components) that enhance phagocytosis (Richards and Endres, 2017). The specialized compartment resulting from membrane invagination around the targeted material is termed phagosome (Niedergang and Grinstein, 2018). Interestingly, the soil-living amoeba *Dictyostelium discoideum* uses molecular mechanisms of phagosome maturation very similar to higher eukaryotic cells, such as macrophages (MFs) (Gotthardt et al., 2002). This efficient “digestive” system of ingested material defines the primary function around which phagocytosis extends its functional ability throughout evolution (Desjardins et al., 2005). However, despite that phagocytosis is often proposed as an evolutionarily conserved mechanism, the diversity and variability of proteins associated with phagosomes across the different eukaryotic species suggest that phagocytosis may have evolved independently several times (Yutin et al., 2009; Mills, 2020).

The kingdom Animalia is composed of multicellular eukaryotic organisms. This cellular scaling has required the acquisition of cell–cell adhesion, communication, cooperation, and specialization (Niklas, 2014). Organism size has always been considered an important factor for the evolution of multicellularity. The advantages of increased size include predator evasion, increased motility, and an increased capacity to store nutrients. Interestingly, the organism size has an impact in cellular specialization, which may evolve more easily in larger organisms (Willensdorfer, 2008). In animals, phagocytosis has extended from the nutritional function to key roles in homeostasis, such as apoptotic cell removal, tissue remodeling, and immune defense (Desjardins et al., 2005). Hartenstein and Martinez have recently reviewed the role of phagocytosis in nutrition and have compared this function of invertebrate enteric phagocytes/enterocytes with MF ability to eliminate pathogens and damaged cells (Hartenstein and Martinez, 2019). Endodermal-derived enterocytes play indeed a prominent role in the invertebrate digestive system by taking up the extracellularly pre-digested material and completing the digestive process intracellularly. By contrast, MFs are mesodermally derived motile cells that engulf and digest foreign materials and cellular detritus that threaten the integrity of the organism. Thus, phagocytosis is an ancient process that likely evolved from the feeding of phagotrophic unicellular organisms to the defense against pathogens in complex organisms. Non-nutritional-related phagocytic cells observed in invertebrate species bear different names (e.g., amoebocytes, coelomocytes, or hemocytes) depending on the hosting species, but basically they have a MF-like appearance and have, to a certain extent, comparable functions as part of the innate immune system (Table 1) (Buchmann, 2014). The hypothesis of a common origin for immunity and digestion is mainly based on the existence of shared components such as enzymes, receptors, signaling pathways, and cellular processes (Broderick, 2015). Thus, many of the enzymes involved in immunity play also a role in digestion (e.g., lysozymes and proteases), with specific contexts for which these functions cannot be distinguished, e.g., for animals that capture and feed on bacteria. However, an extensive transcriptomic analysis done in different phagocytic cell types across widely divergent clades was inconclusive for homology assessments (Hartenstein and Martinez, 2019). Anyhow, in immunity, bacteria internalized via phagocytosis are typically sequestered within phagolysosomes where several antibacterial strategies are used to kill and degrade them, such as compartment acidification, enzyme production and activation, and generation of reactive oxygen species (ROS). Many types of eukaryotes produce ROS, which likely represent an ancient antimicrobial strategy for targeting intracellular bacteria (Richter and Levin, 2019).

**Table 1:** Main features of MF-like cells across species.

	**Diploblastic**	**Platyhelminths**	**Nematoda**	**Arthropoda**	**Echinodermata**	**Vertebrates**			
Representative organism	Cnidaria, Porifera	Planarian	*Caenorhabditis elegans*	*Drosophila melanogaster*	Sea urchin	Zebrafish	Birds	Mouse	Human
Body cavity	No	Acoelomates	Pseudocoelomates	Coelomates					
Gut formation	No	Protostomia			Deuterostomia				
Adaptive immunity	No					Yes			
Phagocyte name	Amoebocytes	Reticular cells	Coelomocytes	Plasmatocytes (different subsets)	Hemocytes/Filopodial cells/Ovoid cells	Macrophages			
Gut-specific population	No			Proventriculus	No	Yes			
Main known functions	Digestive phagocytosis Nutrient transport	Digestive and immune phagocytosis Nutrient transport MORN2-mediated antimicrobial activity	Digestive phagocytosis Fat consumption Life span extension upon starvation Metal detoxification	**Exclusive immune phagocytosis** Efferocytosis Wound healing Antimicrobial signaling IESCs proliferation Glucose homeostasis	Digestive and immune phagocytosis Encapsulation Syncytia formation	**Exclusive immune phagocytosis** Protection against infection and inflammation Microbiota shaping Th17 cells/Tregs regulation Intestinal inflammatory lymphangiogenesis	**Exclusive immune phagocytosis** Antigen uptake Protection Against invading pathogens Antimicrobial activities	**Exclusive immune phagocytosis** Multiple functions depending on their location (see Table 2)	

*IESCs: intestinal epithelial stem cells; Tregs, regulatory T cells*.

Interestingly, phagocytosis shares molecular mechanisms with autophagy, a degradative cellular process in which eukaryotic cells digest their own components (Birgisdottir and Johansen, 2020). Like phagocytosis, autophagy is an ancient highly conserved process likely to date back to the common ancestor of all eukaryotes (Duszenko et al., 2011). Like phagocytosis, autophagy likely evolved from a cellular nutrition mechanism to become a key player in cellular homeostasis and defense against pathogens. Although autophagy and phagocytosis are activated by different mechanisms, they converge on similar pathways that are regulated by shared molecules. Thus, LC3-associated phagocytosis (LAP) involves engulfment of large extracellular particles through the engagement of components of the autophagy machinery among which Beclin 1, the phosphatidylinositol 3-kinase Vps34, ATG (autophagy) family proteins, and finally LC3 (Sanjuan et al., 2007; Martinez et al., 2011, 2015). LC3 recruitment to the phagosome favors phagosome fusion with lysosomes, acidification, and ingested material degradation. LAP is involved in several phagocyte functions, such as pathogen clearance, antigen presentation by major histocompatibility complex (MHC) class II molecules, regulation of proinflammatory cytokine production and efferocytosis (Martinez, 2018).

From its earliest beginnings, the study of innate immunity has greatly benefited from works carried out on simple organisms, starting from the discovery of phagocytosis significance in starfish larva by Elie Metchnikoff to the more recent discovery of PRRs in the fruit fly by Jules Hoffman (Lemaitre et al., 1996; Hoffmann and Reichhart, 2002; Buchmann, 2014; Gordon, 2016). Indeed, these organisms combine easy genetic manipulations and phenotypic analyses with fast generation renewal and simplified cell diversity and signaling pathways including key elements conserved across species. It is therefore important to appreciate the diversity of MFs across species to have a complete picture of them. With the tissue organization of complex organisms, MFs have acquired new functions within their residence niche where they maintain strong relationships with their neighboring cells, allowing their resident tissue to function properly. In this review, we describe the nature of MFs and MF-like cells across the animal kingdom with a special focus on the intestinal tissue of each species when data are available. We consider more precisely the local and regional specialization of MFs in the mammalian intestine and discuss recent findings highlighting their great diversity of functions from one location to another.

## Macrophage-Like Cells of the Diploblasts

**Figure 1:**
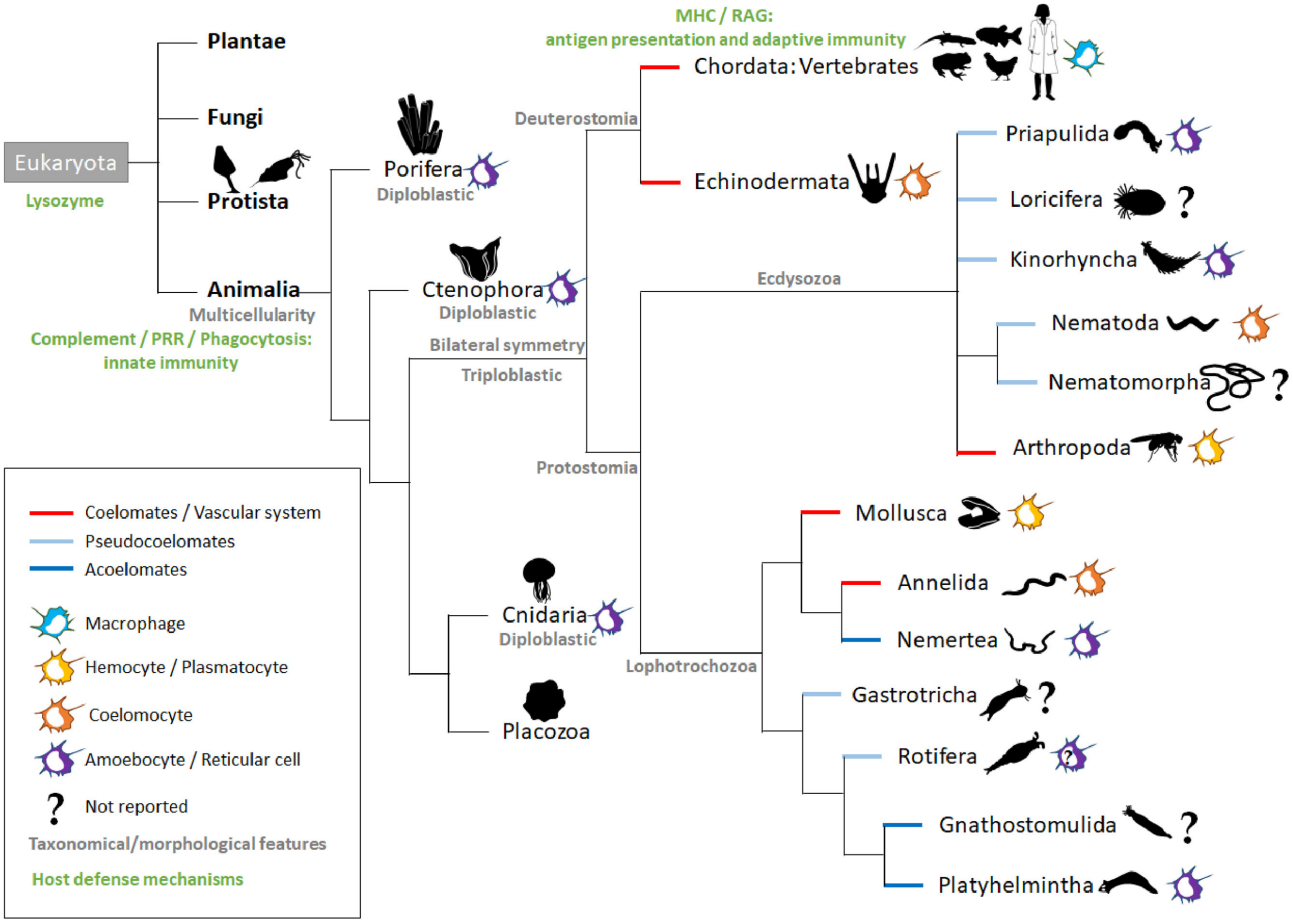
Intestinal macrophage-like cell types across animal species. The main kingdoms (bold black) of the Eukaryota domain are listed. In animals, the representative phyla are named and ordered according to currently used phylogenetic trees (Peterson and Eernisse, 2016; Giribet and Edgecombe, 2017; Kocot et al., 2017). Additional information is added in gray (taxonomical or morphological) in green (host defense mechanisms; nutritional phagocytosis not taken into account) and by the colored lines (embryological development). For each phylum, colored icons on the right symbolize the macrophage-like cell type; the question marks indicate a lack of literature for the phylum. In Rotifera, amoebocytes have been described, but their function is more related to their motility, and there are no reports related to their immunological role (Baumann et al., 2000). MHC, Major Histocompatibility complex; PRR, pattern recognition receptors; RAG, recombination-activating gene.

Living cells depend on a constant supply of energy-rich organic molecules from the environment, making the emergence of a specialized system for food digestion and nutrient absorption a crucial innovation for multicellular organisms. The most ancient division within the animal kingdom is between diploblasts and triploblasts (Figure 1). Diploblasts are radially symmetrical animals with two distinct germ layers: an inner layer or endoderm/gut and an outer layer or ectoderm/skin. In between these two layers, triploblasts have an additional layer: the mesoderm (Telford et al., 2015). Because of the lack of this intermediate layer, mesodermal MFs *per se* are not found in diploblasts. Instead, the gelatinous matrix (mesoglea) between both layers contains large numbers of motile amoebocytes that carry out multiple functions, the most primitive being digestion (Table 1). Amoebocytes ingest and digest food caught by enterocytes and transport nutrients to the other cells. Amoebocytes have been reported in the different diploblastic phyla: Cnidaria (Menzel et al., 2015), Ctenophora (Traylor-Knowles et al., 2019), and Porifera (Adamska, 2016). However, in Placozoa, a sister phylum of Cnidaria, amoebocytes have not been described, probably because these animals are mostly composed of epithelial cells (Mayorova et al., 2019). The ability to phagocyte and move in the mesoglea makes the amoebocytes very similar to mesodermal MFs. Additionally, the presence in these animals of conserved innate defense mechanisms, such as PRRs and pore-forming proteins (e.g., the MF-expressed gene 1 protein, Mpeg1), supports the participation of these amoebocytes in innate immunity (Brennan and Gilmore, 2018; Walters et al., 2020).

## Macrophage-Like Cells in Acoelomate and Pseudocoelomate Protostomes

The triploblasts have two major branches, the Protostomia and Deuterostomia (Figure 1). Their names reflect the fundamentally different fates of the blastopore, the primary embryonic gut opening (Nielsen et al., 2018). In protostomes, the blastopore forms the mouth with the anus forming secondarily (protostomy = mouth first); in the deuterostomes, it is the other way around (deuterostomy = mouth second). The presence of a mouth creates an asymmetry with an anterior–posterior axis making the triploblastic condition a synonym of Bilateria. The gastrointestinal tract (GIT) displays diversified levels of complexity according to species, with the endoderm-derived one-way gut of most bilaterians being the prevailing and more specialized form (Annunziata et al., 2019).

The majority of invertebrates belong to Protostomia, whereas all vertebrates and few invertebrates belong to Deuterostomia (Figure 1). During embryonic development, if a split in the mesoderm forms a fluid-filled body cavity termed coelom, the animal is referred to as coelomate. When the space between the ectoderm and endoderm tissue layers is filled with a meshwork of mesodermal cells (or parenchyma), the animal is referred to as acoelomate. When the mesoderm has fluid-filled clefts in this meshwork, the animal is then termed pseudocoelomate (Monahan-Earley et al., 2013). Acoelomates and pseudocoelomates are found only in Protostomia (Figure 1). By contrast, coelomates are found in both lineages. In several invertebrate phyla, motile MF-like cells in the parenchyma or coelom take up cellular debris resulting from dying cells and actively distribute digested foodstuffs, receiving this material from enteric phagocytes lining the gut (Hartenstein and Martinez, 2019).

The acoelomate protostomes obtain their oxygen and food by simple diffusion across the skin and gut and throughout the intercellular medium. Freely moving reticular cells have been observed in the parenchyma of the platyhelminths (flatworms) (Morita, 1995). These reticular cells are mesenchymal cells that play an important role in nutrient transportation and phagocytosis of foreign material, acting as an immune surveillance system (Table 1). Planarian platyhelminth antimicrobial activities involve an orthologous protein for MORN2, which has been associated with LAP and resistance to bacterial infection in human MFs (Abnave et al., 2014). In Nemertea, amebocytes arising from the intestinal segment were reported to play a central role in graft rejection (Langlet and Bierne, 1984). To our knowledge, there are no reports on the presence of MF-like cells in gnathostomulids.

In pseudocoelomates, the pseudocoelomic fluid serves as the circulatory system for nutrients that are taken up, ingested, degraded, and secreted into the pseudocoelom by the intestinal cells. We did not find any report of MF-like cells in the phyla Gastrotricha, Nematomorpha, and Loricifera. However, in Rotifera, the pseudocoelom of several taxa contains free motile amoeboid cells, but so far, no immune function has been reported for these cells (Baumann et al., 2000). By contrast, phagocytically active amoebocytes have been observed in Priapulida (Mattisson and Fänge, 1973) and Kinorhyncha (Neuhaus and Higgins, 2002). For Nematoda, the cells contained in the pseudocoelomic fluid are termed coelomocytes. *Caenorhabditis elegans* is a simple and genetically tractable nematode model that has enabled key advances in immunity (Willis et al., 2020). However, there is no evidence that their coelomocytes provide a potent defense against bacterial infection (Table 1). These six oblong MF-like scavenger cells located in the *C. elegans* body cavity are indeed dispensable to the viability and survival of the worm (Fares and Greenwald, 2001). Nevertheless, studies of *C. elegans* coelomocytes identified novel components of the endocytic machinery that are conserved in mammals (Fares and Greenwald, 2001; Sato et al., 2014). Moreover, *C. elegans* coelomocytes have been shown to regulate fat consumption and life span extension upon starvation (Buis et al., 2019). Finally, they participate in metal detoxification (Tang et al., 2020). Interestingly, old studies performed in another nematode, *Ascaris suum*, have documented the encapsulation of bacteria by coelomocytes (Bolla et al., 1972).

## Macrophage-Like Cells in Coelomate Protostomes With an Open Circulatory System

The advantage of a true coelom is the ability of the inner mesenteric layer to suspend the central gut in the middle of the animals, allowing them to increase their body size. In addition, a circulatory system helps size increase by reducing the functional diffusion distance of nutrients, gases, and metabolic waste products. In animals that have evolved coelom along with a vascular system, cells with the characteristics of MFs are prominent among the circulating cells, commonly referred to as coelomocytes or hemocytes (Hartenstein, 2006). During development, they represent the professional MFs that eliminate apoptotic cells. In addition, they cooperate with humoral factors to battle invading parasites and microbes, many of which enter through the digestive tract.

Blood vascular systems follow one of two principal designs: open or closed. In open circulatory system, the blood, referred to as hemolymph, empties from a contractile heart and major supply vessels into the body cavity termed hemocoel, where it directly bathes the organs. This occurs in arthropods and non-cephalopod molluscs.

In Arthropoda, the fruit fly *Drosophila melanogaster* has been widely used as a suitable model to study innate immunity and has provided invaluable contributions to the knowledge of innate immune system signaling pathways (Hoffmann and Reichhart, 2002). In *D. melanogaster*, hematopoiesis does not occur in adult but only during development through two waves (Wood and Martin, 2017; Banerjee et al., 2019; Sanchez Bosch et al., 2019). The first wave occurs in the embryo and gives rise to hemocytes that proliferate during the larval stages. The second wave of hematopoiesis occurs at the larval stage in an organ called the larval lymph gland. MF-like cells termed plasmatocytes represent about 95% of the total hemocyte population in adult. A single-cell transcriptome of hemocytes made it possible to characterize different subsets of plasmatocytes (ranging from 4 to 12 depending on the study), showing an interesting parallel with the great diversity of MFs in mammals (Cattenoz et al., 2020; Cho et al., 2020; Fu et al., 2020; Tattikota et al., 2020). Although the precise functions of each of these subsets remain to be established, plasmatocytes globally serve essential roles in immune response to infection and wound healing (Table 1). While lack of plasmatocytes does not impair fruit fly development, it indeed induces a strong susceptibility to infections by various microorganisms, due notably to an absence of phagocytosis in deficient fruit flies (Charroux and Royet, 2009). Plasmatocytes do not only patrol the body in the circulation but also associate with specific tissues, such as the intestinal epithelium. In the *D. melanogaster* model, the intestine is composed of three main parts: the foregut, the midgut, and the hindgut. The fore- and hindgut have an ectodermal origin, whereas the midgut, which is the functional equivalent of the mammalian small intestine (SI), has an endodermal origin. Plasmatocytes of embryonic origin specifically colonize a region at the foregut/midgut junction known as the proventriculus, where they form a discrete group of functional MFs able to phagocytose both apoptotic bodies and bacterial intruders (Charroux and Royet, 2009; Zaidman-Rémy et al., 2012). Plasmatocytes circulating in the hemolymph can also infiltrate the midgut when necessary (Ayyaz et al., 2015). In addition to their phagocytic activity, plasmatocytes relay intestinal infection-induced oxidative stress signal and nitric oxide production to the fat body, an organ equivalent to the vertebrate liver, which produces an antimicrobial peptide response (Wu et al., 2012). Like mammal MFs, plasmatocytes switch their metabolic program to aerobic glycolysis in order to mount an efficient antibacterial response (Krejčová et al., 2019). Upon injury, circulating plasmatocytes release the cytokines of the unpaired (Upd) family Upd2 and Upd3, which by retrospective alignments of type I cytokines and functional analogies are most closely related to the vertebrate leptins (Rajan and Perrimon, 2012; Beshel et al., 2017). These cytokines bind to the receptor Domeless that activates the JAK-STAT pathway in the fat body and in the gut, where it stimulates intestinal stem cell proliferation, thereby contributing to fly survival (Chakrabarti et al., 2016).

In bivalve molluscs, the distribution of blast-like cells suggests that hematopoiesis may be widespread in connective tissue, with further development of hemocytes in the hemolymph (Hine, 1999). Two main sub-populations of hemocytes have been identified: granulocytes containing many cytoplasmic granules and hyalinocytes containing few or no granules (Girón-Pérez, 2010). Granulocytes are the main cell type involved in the cellular immune defense of bivalves (Rolton et al., 2020). They are also involved in other physiological functions, such as wound healing and shell repair, digestion, and transport of nutrients. Indeed, in the gut lumen, hemocytes ingest and digest foreign materials and transport the digested materials to the gut lining or other tissues. In addition to this digestive function, hemocytes can engulf and phagocytize foreign pathogens present on the mucosal surfaces of oysters as part of their innate immune functions. Hemocytes routinely traffic between the hemolymph and the outer surfaces of oysters (Provost et al., 2011).

## Macrophage-Like Cells in Coelomate Protostomes With a Closed Circulatory System

Closed circulatory systems occur in a wide variety of invertebrates including annelids, cephalopods, and non-vertebrate chordates. Earthworms, which are the best known of all annelids, belong to the class Oligochaeta. Their gut surface is in permanent contact with ingested soil. Moreover, the nephridia and dorsal pores enable microorganisms to enter the coelomic cavity. Hence, both coelom and gut interact with naturally occurring soil microorganisms and have to face strong antigenic environment (Prochazkova et al., 2020). The free circulating immune cells of the coelomic cavity, termed coelomocytes, can be subdivided into two subpopulations, the eleocytes and the amoebocytes (Engelmann et al., 2016). Eleocytes are highly autofluorescent cells due to their large granules termed chloragosomes that contain riboflavin. Eleocytes originate from the chloragogen tissue surrounding the gut and are considered as the terminal differentiation stage of sessile chloragocytes released from this tissue. They have mainly accessory functions such as maintenance of pH and storage of glycogen and lipids. By contrast, amoebocytes are MF-like cells with a broad range of defense functions, including phagocytosis (Engelmann et al., 2016). Two types of amoebocytes have been described, hyaline and granular amoebocytes, without clear separate functions. PRRs [coelomic cytolytic factor (CCF) and lipopolysaccharide (LPS)-binding protein (LBP)] and the TLR signaling pathway molecule Myd88 genes are typically expressed by amoebocytes but not eleocytes, supporting the role of amoebocytes in pathogen detection and neutralization (Bodó et al., 2018). Moreover, amoebocytes express higher levels of the oxidative stress-related super oxide dismutase and antimicrobial lysozyme and lumbricin genes (Bodó et al., 2018). Dermal contact with immunostimulants decreases coelomocyte total number but increases the proportion of granular amoebocytes among them and induces ROS production (Homa et al., 2013, 2016). Experimental microbial challenge triggers the release of phagocytic coelomocytes from the mesenchymal lining of the coelom and thus increases the defense reaction in the coelomic cavity of earthworms (Dvorák et al., 2016).

## Macrophage-Like Cells in Invertebrate Deuterostomes

Deuterostomes include two main phyla: Echinodermata and Chordata (Figure 1). In echinoderms, the circulating immune cells, i.e., the coelomocytes, are heterogeneous in morphology, size, relative abundance, and functions. This makes a single standard classification for all echinoderms a difficult task. The distribution of these cell types is also highly variable among species and even at the individual level (Smith et al., 2018). Nevertheless, phagocytes are present in all echinoderm classes and are the main effectors of the echinoderm immune system. These phagocytes respond to immune challenges through phagocytosis, encapsulation, syncytia formation, and expression of complement components (Golconda et al., 2019).

The sea urchin larva has five major types of immune cells that populate the body cavity (blastocoel), including two phagocytic cell types termed filopodial and ovoid cells (Table 1) (Ho et al., 2017). Filopodial cells extend long filopodia that form a reticular network in the blastocoel (Buckley and Rast, 2019). They are likely the MF-like cells observed by Elie Metchnikoff in his seminal works on phagocytosis. Ovoid cells are rarely present at steady state but rapidly appear upon immune challenge and could therefore represent an activation state of some of the filopodial cells. Upon sea urchin larva gut disturbance through the presence of pathogenic bacteria in the seawater, a coordinated immune response takes place (Ho et al., 2017). A subset of immune cells termed pigment cells rapidly migrates from the ectoderm to the gut epithelium where they secrete their antibacterial iron chelator pigment echinochrome A (Ho et al., 2017; Coates et al., 2018). Then, the number and duration of cell–cell interactions among immune cells and with the gut epithelium increase (Ho et al., 2017). Finally, filopodial cells quickly phagocytose bacteria that penetrate the blastocoel of larvae. This coordinated immune response is at least in part launched by secretion of IL-17 family members by gut epithelial cells (Buckley et al., 2017).

## Vertebrate Macrophages

The phylum Chordata consists of three subphyla: Urochordata, Cephalochordata, and Vertebrata. Vertebrates possess non-phagocytic enterocytes, and a clear dichotomy is made at this level between the digestive and immune function of phagocytosis (Hartenstein and Martinez, 2019). Moreover, vertebrates have evolved adaptive immunity that can recognize and respond to specific antigen determinants thanks to the somatic DNA rearrangement of segmental elements encoding the antigen binding regions of their T and B cell receptors (Cooper and Alder, 2006). Together with adaptive immunity appears a new type of mononuclear phagocytes termed dendritic cells (DCs). DCs make the link between innate and adaptive immunity by initiating and controlling antigen-specific immunity through presentation of antigenic epitopes on MHC class I and class II molecules (Banchereau and Steinman, 1998). Therefore, the vertebrate mononuclear phagocyte system comprises monocytes, MFs, and DCs, as well as their lineage-committed progenitors (Guilliams et al., 2014). The intestinal immune system of vertebrates comprises a unique array of innate and adaptive immune cells. Along the intestinal tract, immune cells are either disseminated throughout the mucosa forming a diffuse distribution or clustered in organized lymphoid tissues. The latter, termed organized gut-associated lymphoid tissues (GALT), initiate the intestinal immune response. Organized GALT have been reported in their simplest forms in all classes of vertebrates but are especially well-developed in the endotherms, mainly mammals and birds.

The bone marrow is the hematopoietic organ in all vertebrates except some amphibians in which hematopoiesis can also occur in the liver and fishes in which hematopoiesis occurs only in the kidney. Fishes are the most primitive animals in which an adaptive immunity is present. In zebrafish, the first embryonic MFs originate from the mesoderm and migrate over the yolk ball before colonizing other tissues, whereas in adults, myeloid-lineage progenitors arise from the kidney (Stachura and Traver, 2011). MFs and DCs are especially abundant in the spleen and gut (Wittamer et al., 2011). In the adult zebrafish, the gut can be divided following the anterior–posterior axis into seven segments, from the proximal S1 to the most distal S7 (Wang et al., 2010). Each segment exhibits functional differences and also similarities to the mammalian GIT; e.g., the S7 represents the colon-like region (Wang et al., 2010; Lickwar et al., 2017). Distribution of MFs and DCs along these segments and the ability of these phagocytes to sample luminal antigens depending on their location have not been determined so far. Interestingly, Interferon Regulatory Factor 8 (IRF8) depletion leads to a lack of MFs during embryonic development with only partial recovery in adults (Li et al., 2011; Shiau et al., 2015; Ferrero et al., 2020). Thus, brain and gut resident MFs remain strongly impacted by IRF8 deficiency (Earley et al., 2018). Importantly, like in mammals, intestinal MFs are required for shaping the gut microbiota, and dysbiosis occurs in MF-deficient zebrafish (Table 1) (Earley et al., 2018). In addition, IRF8-dependent MFs are the main producers of the complement C1q genes in the intestine (Earley et al., 2018). Zebrafish intestinal MFs show other similarities with their mammal counterparts. Thus, like in mouse and human, CD4^+^ MFs and regulatory T (Treg) cells reside in the zebrafish gut mucosa (Dee et al., 2016). Moreover, microbiota and inflammation promote G-protein-coupled receptor 35 expression in mouse and zebrafish intestinal MFs, which have a protective role during intestinal inflammation by inducing TNF synthesis upon lysophosphatidic acid binding (Kaya et al., 2020). Finally, like in mammals, intestinal inflammation entails infiltration into the gut mucosa of inflammatory MFs, which elicit a Th17 cell response together with a decrease of Treg cells (Coronado et al., 2019). Moreover, in a zebrafish colitis model, MFs promote intestinal inflammatory lymphangiogenesis via their vascular endothelial growth factor gene expression (*vegfa, vegfc*, and *vegfd*), highlighting the potential of the zebrafish model to investigate the mechanism of lymphangiogenesis in inflammatory bowel diseases (IBDs) (Okuda et al., 2015).

Amphibians rely heavily on MFs not only for immune defense but also for homeostasis and tissue remodeling/resorption. Most of the literature on amphibian MFs is related to programmed cell death and tissue remodeling during metamorphosis (Grayfer and Robert, 2016). Hematopoiesis of primitive and mainly aquatic amphibian species occurs in the liver, whereas hematopoiesis of more terrestrial amphibian species occurs in the bone marrow (Grayfer and Robert, 2016). In the frog *Xenopus laevis*, the principal site of hematopoiesis is still the liver subcapsular region, but myelopoiesis, i.e., the differentiation of the granulocyte MF precursor (GMP) into granulocytes and MFs, occurs in the bone marrow (Grayfer and Robert, 2013; Yaparla et al., 2016). Precursors with GMP potential migrate from the liver to the bone marrow under the influence of chemokines enriched in the bone marrow, such as CXCL12 (Yaparla et al., 2019). MF differentiation is controlled through binding of the main MF growth factor, colony-stimulating factor-1 (CSF1) to its CSF1 receptor (CSF1R), which is almost exclusively expressed on committed MF precursors (Grayfer and Robert, 2016). IL-34 is as an alternative CSF1R ligand, giving rise to morphologically and functionally distinct MFs (Yaparla et al., 2020). Unfortunately, to our knowledge, the literature on amphibian intestinal MFs consists mainly of old descriptive studies. Lymphoid aggregates resembling mammalian isolated lymphoid follicles (ILFs) have been observed in the urodele amphibians (Ardavín et al., 1982). In these structures, the number of goblet cells decreases, and lymphoid cells, including MFs, penetrate the intestinal epithelium. In the gut lamina propria of toads, MFs tend to cluster and interact with lymphocytes and plasma cells (Chin and Wong, 1977).

Most studies on reptile immune function have focused on systemic immune responses, leaving an important knowledge gap in the mucosal immune responses. Indeed, literature on reptile intestinal immunity consists mainly of descriptive studies (Borysenko and Cooper, 1972; Zapata and Solas, 1979; Solas and Zapata, 1980; Ashford et al., 2019). Reptiles possess primary lymphoid organs such as bone marrow and thymus but lack secondary lymphoid tissues such as Peyer's patches (PPs) or mesenteric lymph nodes (MLNs). Instead, numerous ILF-like lymphoid aggregates are located throughout the small and large intestines. These aggregates are enriched in small lymphocytes and MFs (Borysenko and Cooper, 1972; Zapata and Solas, 1979; Solas and Zapata, 1980; Ashford et al., 2019). MFs are dispersed in the lamina propria but can migrate to the intestinal epithelium in these lymphoid aggregates (Solas and Zapata, 1980). Interestingly, the number of lymphoid aggregates in the SI of poikilothermic snakes depends on the season, diminishing in spring and summer (Solomon et al., 1981). Moreover, temperature can affect functions of lizard MFs, which have an optimal phagocytic activity at 25°C (Mondal and Rai, 2001). In reptiles, the enteropathogenic bacteria *Salmonella enterica* colonize the intestinal tract without any signs of disease, but MFs seem not to be involved in this protection since *S. enterica* is able to kill turtle MFs (Pasmans et al., 2002).

Like mammals, birds have a well-developed mucosal immune system, with several organized GALT. They include the primary lymphoid organ termed bursa of Fabricius and several secondary lymphoid organs, namely, PPs, cecal tonsils, and Meckel's diverticulum (Casteleyn et al., 2010). The chicken gut lamina propria contains various innate immune cells such as heterophils (the avian polymorphonuclear cells), natural killer cells, DCs and MFs, although the differences between the latter two have not been carefully assessed (Broom and Kogut, 2018). Chicken MFs/DCs display a range of PRRs, expression of MHC class II, and phagocytic and antimicrobial activities. Like in mammals, early-life microbial colonization is critical for the immunological maturation of the avian gut, and short early-life antibiotic treatment induces alteration of mucosal gene expression and a decrease of MF number in the gut lamina propria for at least 2 weeks (Schokker et al., 2017). In chicken duodenum, jejunum, and ileum, MFs/DCs are involved in antigen uptake and provide protection against invading pathogens (Table 1) (Taha-Abdelaziz et al., 2020). During coccidiosis, MFs are the principal cell type involved in the clearance of the sporozoites by phagocytosis (de Geus and Vervelde, 2013). During *Salmonella* infection, resistance depends on a genetic locus *SAL1* and has been linked to MFs with better oxidative killing activity and greater and faster expression of proinflammatory cytokines (Wigley, 2014). During dextran sulfate sodium (DSS)-induced intestinal inflammation, increased monocyte/MF infiltration occurs in all segments of laying hen intestine (Nii et al., 2020).

In mammals, embryonic MFs initially originate from yolk sac erythro-myeloid progenitors (Hoeffel and Ginhoux, 2018). Subsequently, early hematopoietic stem cells (HSCs) settle first in the fetal liver and later in bone marrow to form a permanent, self-renewing source of monocytes. Monocytes infiltrate tissues and can replace and differentiate into tissue-resident MFs to varying degrees depending on the organs and the encountered immune challenges, with most MFs keeping an embryonic origin and self-renewal (Hoeffel and Ginhoux, 2018; Liu et al., 2019). Importantly, MF renewal by monocytes is especially prevalent in the intestine, which is always subject to antigenic stimulation whether through food, drink, or microbiota (Bain et al., 2014). In rodents and in human, the GIT is complex and divided longitudinally from the duodenum to the rectum, with functional and morphological distinctions between the small (duodenum, jejunum, and ileum) and large (cecum and proximal and distal colon) intestines (Mowat and Agace, 2014). The SI is specialized in the absorption of nutrients, whereas the primary function of colon is the absorption of water and electrolytes. The SI has villi that increase its surface of exposure to the intestinal lumen content, a thinner and less well-organized mucus layer, and reduced microbial communities than the colon. Mammalian MFs can fulfill the auxiliary functions necessary for the homeostasis of each tissue of residence, such as peristaltic movements and tolerance induction toward dietary and microbiota-derived antigens in the intestine. In the recent years, single-cell RNA sequencing associated with high-resolution confocal microscopy, multiparameter flow cytometry, and functional assay analyses have allowed to reveal unsuspected aspects of the local and regional specialization of MFs in mouse and human intestine as discussed below and shown in Table 2.

**Table 2:** Local and regional specialization of intestinal macrophages in human and mouse.

		**Location**	**Name**	**Main neighbors**	**Main functions**	**Species**	**Key markers**	**References**
Radial specialization	Effector sites: Villus and colonic MFs	Subepithelial SI and colon	LPM	IECs fibroblastic cells Immune cells	Antigen sampling Tissue protection and repair Treg and Th17 cell equilibrium	Mouse Human	CD64, CX_3_CR1, F4/80, MerTK CD14, CD16, CD64, MerTK, CD163	Tamoutounour et al., 2012; Bain et al., 2013 Bujko et al., 2018
		Perivascular SI and colon	Perivascular MF	Endothelial cells	Regulation and strengthening of the vasculature Bacterial translocation blockade Systemic antigen uptake	Mouse	CX_3_CR1, CD169, CD206	De Schepper et al., 2018; Honda et al., 2020; Kang et al., 2020
		Crypt base SI and colon	Crypt base-associated MF	Lgr5^+^ epithelial stem cells Paneth cells	Maintain the renewal of the SI crypts Promote the crypt regenerative response Inflammatory monocyte recruitment	Mouse	CSF1R/CD115, CD169, CD206	Pull et al., 2005; Asano et al., 2015; Sehgal et al., 2018; Kang et al., 2020
		Submucosa/muscularis	Muscularis MF	Enteric neurons Smooth muscle cells	Smooth muscle contraction and Peristaltism Tissue-protective	Mouse Human	CX_3_CR1, MHC-II, BPM2, β2 AR CD14, CD11b, CD209	Muller et al., 2014; Gabanyi et al., 2016; De Schepper et al., 2018 Bujko et al., 2018
	Inductive sites: Peyer's patch MFs	Subepithelial dome Upper follicle	TIM-4^−^ LysoMac	FAE/M cells RANKL^+^ stromal cells DCs, B and T cells	Uptake of particulate antigens Innate defense Apoptotic IEC removal	Mouse Human	MerTK, CX_3_CR1, lysozyme, CD4 Lysozyme, CD11c, SIRPα	Bonnardel et al., 2015 Wagner et al., 2020
		Interfollicular regions Lower follicle	TIM4^+^ LysoMac	T cells	Apoptotic T cell removal	Mouse Human	MerTK, CX_3_CR1, CD4, TIM-4 Lysozyme, TIM-4	Bonnardel et al., 2015 Wagner et al., 2020
		Germinal center	Tingible body MF	B cells	Apoptotic B cell removal	Mouse Human	MerTK, CX_3_CR1, CD4, TIM-4 Lysozyme, TIM-4	Bonnardel et al., 2015 Wagner et al., 2020
		Submucosa/muscularis externa	Serosal/muscularis MF	?	?	Mouse Human	MerTK, CX_3_CR1, TIM-4 Lysozyme, TIM-4, CD163	Bonnardel et al., 2015 Wagner et al., 2020
Gut segment specialization	Antigen-inducible sampling mechanisms	SI lumen	Luminal MF	IECs	Immune exclusion by pathogen capture (*Salmonella* Typhimurium)	Mouse	CD11c, CX_3_CR1, F4/80	Man et al., 2017
		Distal ileum	TED^+^ LPM	IECs	Pathogenic bacteria uptake (*Salmonella* Typhimurium)	Mouse	CX_3_CR1, CD11c, CD11b	Niess et al., 2005
		Peyer's patches	TMD^+^ LysoDC	M cells	Pathogenic bacteria uptake (*Salmonella* Typhimurium)	Mouse	CX_3_CR1, lysozyme, CD11c	Lelouard et al., 2012; Bonnardel et al., 2015; Wagner et al., 2020
		Distal colon	BLPs^+^ LPM	IECs	Fluid sampling and fungus toxin detection IECs protection by regulating fluid absorption	Mouse	CD11c, CD11b, MHCII, F4/80, CX_3_CR1, CD64	Chikina et al., 2020
	Adaptive immune responses	Distal ileum	LPM	?	Antigen-specific Th17 cells induction in response to SFB ileum colonization	Mouse	CD64, CSF1R	Panea et al., 2015
		Colon	LPM	?	Induction of Th17 cells and antibodies in response to fungus colon colonization Immune surveillance and maintenance of a balanced colonic fungal community	Mouse Human	CX_3_CR1	Leonardi et al., 2018

*SI, small intestine; MF, macrophages; LPM, lamina propria MF; DC, dendritic cells; IECs, intestinal epithelial cells; FAE, follicle-associated epithelium; TED, trans-epithelial dendrite; TMD, trans-M cell dendrite; BLP, balloon-like protrusion; SFB, segmented filamentous bacteria; CSF1R, colony-stimulating factor-1 receptor; β2 AR, β2 adrenergic receptor; ?, unknown*.

## Tell Me Where You Live and I Will Tell You What Kind of Macrophage You Are

We have seen that across species, specialized cells assume at least one of the main activities of what we call MF in vertebrates: phagocytosis. These cells show a remarkable plasticity according to their local microenvironment, i.e., the network of factors and cells with which they interact. With the increasing complexity of tissue functions occurring during the metazoan evolution, these cells have diversified even more and have acquired dedicated functions to offer protection and to sustain activity of their tissue of residence.

The specific stromal microenvironment that surrounds HSCs in the bone marrow has been identified decades ago as niches indispensable for the maintenance and differentiation of HSCs (Morrison and Scadden, 2014). However, it is only very recently that this concept of niche has been fully appreciated for peripheral tissues, especially for MF identity imprinting (Gosselin et al., 2014; Lavin et al., 2014; van de Laar et al., 2016). Two recent reviews have described how this local microenvironment is now crucial to be considered in order to better characterize and understand the functions of tissue-resident MFs (Blériot et al., 2020; Guilliams et al., 2020). Nowadays, studies dedicated to the conditioning of MFs by their local microenvironment are mainly developed in human and mouse in which a unique MF transcriptional program seems to correspond to each specific niche of the intestine (Bujko et al., 2018; De Schepper et al., 2018; Kang et al., 2020). Since phagocytic cells interact with and react to external factors and neighboring stromal and immune cells whatever the species considered, we however assume that this concept can be widely extended to other species.

In the following parts, we detail how intestinal MF identity and functions are impacted by their niche of residence. We particularly focus on the (re)categorization of the MFs according to their anatomical location within the intestinal mucosa (Table 2). We also consider the strong influence of two exogenous factors inseparable from the intestine, the intestinal microbiota and the dietary antigens.

### A (Re)Categorization of Intestinal Macrophages by Their Radial Distribution and Local Compartmentalization: Protecting and Supporting Your Immediate Neighbors

Most intestinal MFs along the GIT share some common functions, such as the phagocytosis of microorganisms and dead cells. They also share some common specific markers, such as CX_3_CR1 and F4/80 in mouse and CD14 and CD16 in human (Bain et al., 2013; Bujko et al., 2018). In addition, in both species, intestinal MFs express CD64, CD163, and MerTK. At the exception of immune inductive sites such as PPs as discussed later, gut MFs display anti-inflammatory properties at steady state. They indeed weakly respond to many different innate stimulations, constitutively express IL-10 and its receptor, participate in Treg cell expansion, and protect from colitis (Hadis et al., 2011; Bain et al., 2013; Shouval et al., 2014; Zigmond et al., 2014). According to their anatomical location, intestinal MFs interact with specific cells and detect and respond to specific factors that make them crucial support units of their microenvironment. In turn, the latter is decisive for MF recruitment and differentiation in relation to the different anatomic layers of the intestine. Therefore, from the serosa to the epithelium, MFs are territorialized to accomplish specific functions (Table 2).

Two main categories of MFs are present in the small and large intestines of mammals: *lamina propria* MFs (LPM) and muscularis MFs (MM) (Hume et al., 1984; Mikkelsen, 1995). LPM can be further subdivided into mucosal and submucosal LPM with different life span, transcriptional program, and functions (De Schepper et al., 2018). Mucosal LPM from the SI to the colon line the intestinal epithelium on the one hand and the vasculature on the other hand (Niess et al., 2005; Chieppa et al., 2006; Chikina et al., 2020; Honda et al., 2020). Mucosal subepithelial LPM are thus strategically positioned to sample luminal antigens and to protect the mucosa from enteropathogens that can penetrate the epithelial barrier. In the mouse SI, the pathogenic bacteria *Salmonella* Typhimurium induce the formation of paracellular transepithelial dendrites by subepithelial LPM, allowing them to capture bacteria directly from the lumen (Niess et al., 2005; Chieppa et al., 2006). In accordance with their bacteria-inducible nature, LPM transepithelial dendrites occur more frequently where bacteria are abundant, i.e., at the tip of villi of the terminal ileum rather than in other parts of the SI (Niess et al., 2005; Chieppa et al., 2006). Whether LPM transepithelial dendrites represent an important mechanism of antigen sampling that could occur in absence of pathogenic bacteria remains under debate (McDole et al., 2012). *Salmonella* Typhimurium induce also the migration of mouse subepithelial LPM into the gut lumen to participate in the immune exclusion of the bacteria from the gut (Arques et al., 2009; Man et al., 2017). Importantly, both transepithelial dendrites and luminal migration are dependent on CX_3_CR1 expression by subepithelial LPM and on MyD88-dependent TLR signaling by intestinal epithelial cells (Chieppa et al., 2006; Arques et al., 2009; Man et al., 2017). Whether these partial (dendrites) or complete (luminal migration) transepithelial passages occur in human has yet to be established, especially since CX_3_CR1 expression by human intestinal LPM is reduced as compared with that in mice (Bujko et al., 2018). Nevertheless, a missense mutation in the *CX3CR1* gene has been identified in Crohn's disease patients and was linked to an inefficient antifungal response (Leonardi et al., 2018). This suggests that CX_3_CR1 is at least important in human for the control of the fungi gut community. Unlike conventional DC, mucosal LPM are unable to migrate into the gut-draining MLNs to present pathogen-derived antigens and prime naïve T cells (Schulz et al., 2009; Bravo-Blas et al., 2019). Nevertheless, they can transfer antigens via gap junctions to neighboring conventional DC that can in turn express CCR7, migrate to the MLNs, and prime naïve T cells (Mazzini et al., 2014). Mucosal conventional DC can also acquire soluble and particulate antigens directly from the lumen through several mechanisms (McDole et al., 2012; Farache et al., 2013), raising the question of the real contribution of MFs to the provision of antigens for antigenic presentation.

Another subpopulation of CX_3_CR1^+^ mucosal and also submucosal LPM is closely associated to the intestinal vasculature in mice (De Schepper et al., 2018; Honda et al., 2020). Perivascular LPM are either self-maintaining throughout adulthood, especially submucosal ones, or replaced by monocytes on a regular basis, especially mucosal ones (De Schepper et al., 2018). The full maturation of the latter from Ly6^hi^ monocytes is ensured by the microbiota and by the transcription factor NR4A1, a master regulator of the conversion of CCR2^hi^CX_3_CR1^int^Ly6C^hi^ into CCR2^lo^CX_3_CR1^hi^Ly6C^lo^ monocytes (Honda et al., 2020). Mucosal perivascular LPM form tight interdigitating connections around all of the vasculature of both SI and colon by which they prevent bacteria translocation into the blood circulation (Honda et al., 2020). Therefore, both subepithelial and perivascular mucosal LPM are fully equipped to prevent penetration of pathogens through epithelial and vascular barriers, offering thus a double defensive line. By contrast, submucosal perivascular LPM are distant from the lumen, the microbiota, and potential pathogens. They acquire a transcriptional profile in relation to their niche, including angiogenesis-related genes, and are necessary for the repair and strengthening of the vasculature since lack of perivascular LPM disturbs the vasculature morphology and induces particle leakage from the blood (De Schepper et al., 2018).

At the base of SI and colonic crypts, a specific subset of submucosal LPM expressing CD169 is tightly associated with the epithelial stem cell niche (Pull et al., 2005; Hiemstra et al., 2014; Asano et al., 2015; Sehgal et al., 2018) (Table 2). In the SI, depletion of these stem cell niche-associated LPM following CSF1R blockade induces a defect in Paneth cell differentiation and a reduction in LGR5^+^ intestinal stem cell numbers (Sehgal et al., 2018). This leads to reduced epithelial proliferation and imbalanced intestinal epithelial cell ratio, notably favoring goblet cells. Therefore, stem cell niche-associated LPM are crucial in the appropriate differentiation of SI epithelial cells. Surprisingly, in the colon, *Csf1*-deficient *op/op* mice with strong LPM depletion show normal colonic crypt morphology, suggesting that unlike SI, colon stem cell niche-associated LPM are not essential to maintain the colonic stem cell niche (Cecchini et al., 1994). Nevertheless, during injury, the colonic stem cell niche-associated LPM are essential to promote the regenerative response, i.e., the proliferation and the survival of colonic epithelial progenitors (Pull et al., 2005). Similarly, in fruit fly, plasmatocytes induce stem cell proliferation in the intestine in response to wounding via their secretion of Upd2 and Upd3 (Chakrabarti et al., 2016). Therefore, as for mouse stem cell niche-associated LPM, fruit fly plasmatocytes can be tightly associated with the intestinal epithelium and can play a major role in tissue repair, highlighting a highly conserved mechanism of cooperation between phagocytes and gut epithelial stem cells.

In mammals, MM display either bipolar (circular muscle and deep muscular plexus MM) or stellate (serosal and myenteric MM) shapes and are closely associated with smooth muscles and enteric neurons of the muscularis externa, distant from any luminal stimulation (Mikkelsen, 1995; Phillips and Powley, 2012). Accordingly, MM display a gene expression profile associated with tissue protection and neuronal development (Gabanyi et al., 2016; De Schepper et al., 2018). MM play a major role in regulating intestinal peristalsis by producing BMP2 and PGE2, which act on enteric neurons and smooth muscles, respectively (Muller et al., 2014; Luo et al., 2018). In addition, MM play a neuroprotective role by limiting infection-induced neuronal loss through an adrenergic/arginase 1/polyamines axis (Matheis et al., 2020). The development of MM is ensured by CSF1 produced by their associated enteric neurons (Muller et al., 2014). However, other cells (e.g., endothelial cells or interstitial cells of Cajal) can replace enteric neurons since MM are not impacted in mice lacking enteric neurons, as well as in humans in whom the enteric nervous system is absent from the colon (Hirschsprung disease) (Avetisyan et al., 2018). Neuron-associated MFs are also present in the submucosal LP, where they could play a role in the regulation of the intestinal secretion induced by neurons (De Schepper et al., 2018).

From the SI to the colon, from rodents to humans, most mucosal LPM are continuously renewed by blood monocytes (Tamoutounour et al., 2012; Bain et al., 2013, 2014; Bujko et al., 2018). In mice, LPM differentiation from Ly6C^hi^ monocytes is phenotypically characterized by four developmental stages termed monocyte waterfall (Tamoutounour et al., 2012; Bain et al., 2013). Nonetheless, a large part of MM and of the submucosal LPM, especially those associated with neurons and vasculature, appears to be long-lived self-maintained cells and are barely replaced by circulating monocytes (De Schepper et al., 2018; Shaw et al., 2018). These MFs are indeed distant from the gut lumen and thus from microbiota and dietary antigens, the well-known drivers of intestinal MF replacement by circulating monocytes (Bain et al., 2014; Ochi et al., 2016). This may explain their self-maintaining property, which is very similar to that of MFs residing in other tissues (Hashimoto et al., 2013; Yona et al., 2013; Liu et al., 2019). In humans, a recent study went through the characterization of MFs within the upper part of the SI (Bujko et al., 2018). They encompass four well-defined subsets based on marker expression, transcriptional profiles, maturation stage, life span, and location. LPM and MM represent two of these subsets, whereas the other two are related to the monocyte to MF conversion.

## (RE)Categorization of Intestinal Macrophages by Their Gut Segment Location: Influence of Dietary Antigens and of Microbiota

Local compartmentalization of intestinal MFs is broadly similar between the SI and the colon. However, MF numbers are generally higher in the colon than in the SI. Moreover, despite similar differentiation programs and markers, MFs of the SI and of the colon are clearly distinct. Thus, monocytes infiltrating the gut acquire intestinal segment-specific gene expression profiles (Gross-Vered et al., 2020). Their differences are mainly due to the specific functions of each segment of the GIT and to the different exogenous antigens they are exposed to. Thus, ileal MFs display higher expression of genes related to immune reaction and response to challenge than colonic MFs (Gross-Vered et al., 2020). In the following part, we will describe how phenotypically similar MFs can act differently according to their gut segment location.

As mentioned above, the main function of the SI is to absorb nutrients, and its large surface area is continually exposed to important amounts of dietary-derived products. Microbiota density increases drastically from the duodenum to the colon according to gut physico-chemical environment variations (e.g., oxygen and pH levels). Therefore, the colon faces a huge amount of diverse microorganisms (commensal bacteria, archaea, virus, and fungi) and their derived metabolites. The number of goblet cells also increases significantly from the SI to the colon. Consistent with this increased goblet cell frequency, the mammalian colon is protected by thicker and more organized mucus layers than the SI, keeping microorganisms at bay from the epithelium (Johansson et al., 2008; Bergstrom et al., 2020).

Whereas in the colon the microbiota promotes LPM renewal by circulating monocytes and contributes to their functional diversification (Bain et al., 2014; Kang et al., 2020), in the SI, intestinal microbiota is a major factor neither for the control of MF replenishment nor for their IL-10 production (Ochi et al., 2016). Consistently, there is no difference in the number of SI LPM populations between antibiotic-treated and untreated mice. However, dietary factors can directly regulate homeostasis of SI LPM, and a total deficiency in dietary amino acids or the inhibition of the mTOR-mediated amino acid sensing leads to a reduction of IL-10-producing MF number (Ochi et al., 2016). Actually, many other molecules resulting from the degradation of the food, such as vitamins and aryl hydrocarbon receptor (AHR) ligands, are susceptible either directly or indirectly to influence intestinal MF functions in the SI (Mowat and Agace, 2014).

*Drosophila melanogaster* is a good model to study the impact in the gut of imbalanced diets, such as the high-fat and Western diets, or of potential toxic products, such as fried food-derivatives. In the fruit fly, lipid peroxidation products of fried food induce an increase of ROS production and DNA damages in plasmatocytes (Demir and Marcos, 2017). A similar study was recently performed in mouse and confirmed that dietary peroxidized fats induce proinflammatory responses by peritoneal MFs and resident immune cells in PPs (Keewan et al., 2020). With regard to high-fat diet, Woodcock *et al*. showed that lipid-rich diets reduce the life span of *D. melanogaster* and impair its glucose metabolism (Woodcock et al., 2015). This is due to the activation of the JAK-STAT pathway in response to the Upd3 secreted by the plasmatocytes that become foamy, accumulating neutral triglycerides and other lipids in lipid vacuoles. In mouse, Kawano et al. highlighted that high-fat diet induces CCL2 expression by colonic IEC leading to CCR2-dependent proinflammatory MF infiltration in the colon, which results in inflammasome activation in these newly recruited MF, increased intestinal permeability, and glucose metabolism and insulin resistance impairment (Kawano et al., 2016). The western diet includes a high intake of proteins (mainly from animal-derived sources), saturated fatty acids (SFAs), sugar, processed food, and salt, together with a reduced consumption of vegetables, fruits, vitamins, minerals, and ω-3 polyunsaturated fatty acids (PUFAs). SFAs activate proinflammatory response in MFs through the TLR4-NF-κB pathway (Lee et al., 2003). By contrast, specialized proresolving mediators (SPMs) are a large class of signaling molecules that counteract the effect of proinflammatory dietary antigens on intestinal MFs. SPMs are derived from the metabolism of ω-3 PUFA supplied in the diet, giving rise to protectins, resolvins, and maresins. Alternatively, they are produced as eicosanoids (prostaglandin D2 and E2 and lipoxin A4) by immune (including MFs) and non-immune cells (Na et al., 2019). SPMs influence MF differentiation toward a proresolving phenotype. Proresolving MFs dampen Th1 and Th17 responses, re-establish breached epithelial barrier, limit entry of neutrophils to the site of injury, and promote monocyte migration (Na et al., 2019). Thus, protectins and resolving D1 promote resolution of inflammation by increasing MF phagocytosis and suppressing inflammatory MFs in inflammatory diseases (Buckley et al., 2014). In summary, diet is a key element to take into account when studying variations in LPM functions. However, it is important to keep in mind that most of the research studying the interplay between the dietary antigens and the intestinal MFs have been performed *in vitro*. Therefore, more *in vivo* studies will be required to fully address the impact of diet on the different populations of intestinal MFs.

The main function of the colon is to absorb electrolytes and water and also to manage undigested foodstuffs. Through saccharolytic and proteolytic fermentations, the colon microbiota is involved in the catabolism of remaining indigestible food and produces a variety of metabolites in the colon including short-chain fatty acids (SCFAs), which are involved in colonic LPM conditioning. Thus, antibiotic treatments cause colonic LPM to express increased levels of proinflammatory cytokines following microbiota recolonization and to become responsive to LPS stimulation (Scott et al., 2018). Interestingly, supplementation of antibiotics with the SCFA butyrate, whose production is reduced under antibiotic treatment, restores the anti-inflammatory profile and hypo-responsiveness of colonic MFs (Scott et al., 2018). Administration of butyrate also promotes colonic LPM antimicrobial activities, such as lysozyme, calprotectin, and ROS production (Schulthess et al., 2019). Anti-inflammatory and anti-microbial effects of butyrate are mediated via inhibition of histone deacetylase 3, thus regulating MF transcriptional and metabolic program (Chang et al., 2014; Schulthess et al., 2019). More generally, microbiota contributes to the functional diversification of colon MFs (Kang et al., 2020). It supports in particular colonic LPM production of IL-10 and limits Th1 cell response while promoting Treg cell expansion (Kim et al., 2018).

Colonic LPM conditioning depends not only on microbiota but also on TGFβ and, above all, IL-10 signaling (Schridde et al., 2017; Biswas et al., 2018). Indeed, IL-10 signaling pathway promotes WASP and DOCK8 interaction leading to STAT3 phosphorylation and anti-inflammatory MF polarization (Biswas et al., 2018). By contrast, lack of IL-10 signaling induces a proinflammatory profile on colonic MFs highlighted by IL-23 and IL-1β production, leading to recruitment of Th17 cells and promoting colitis (Shouval et al., 2016; Bernshtein et al., 2019). Loss of IL-10 receptor signaling in mouse and human MFs indeed induces spontaneous colitis and severe infant-onset IBD, respectively (Shouval et al., 2014; Zigmond et al., 2014). Surprisingly, though more inflammatory, these MFs show defect in *Salmonella* Typhimurium killing due to prostaglandin E2 overproduction (Mukhopadhyay et al., 2020).

Depending on the location of encountered microorganisms, CX_3_CR1^+^ LPM induce regionalized antigen-specific Th17 responses (Table 2). Thus, CX_3_CR1^+^ LPM are involved in the induction in the SI of a specific and robust Th17 response against segmented filamentous bacteria (SFB) that colonize specifically the ileum (Panea et al., 2015). Unlike pathogen-elicited Th17 cells that are highly glycolytic inflammatory effector cells producing IFNγ, SFB-induced Th17 cells are non-inflammatory homeostatic tissue resident cells (Omenetti et al., 2019). By contrast, colonization with the fungus *Candida albicans* induces a strong Th17 response in the colon where it resides but not in the SI (Leonardi et al., 2018). Actually, colonic LPM are fully equipped to efficiently recognize and respond to the important fungal communities (mycobiota) found in the distal colon, notably via the C-type lectin receptors dectin-1, dectin-2, and mincle (Iliev et al., 2012; Leonardi et al., 2018). Dectin-1 promotes a proinflammatory program in colonic MFs, resulting in inflammasome-dependent IL-1β secretion and inflammatory monocyte recruitment to the inflamed colon (Rahabi et al., 2020). In contrast, Treg cells regulate the inflammatory properties of colonic MFs by inhibiting their IL-1β and IL-23 production (Bauché et al., 2018). This inhibition involves MHC class II engagement by latent activation gene-3 (LAG-3) expressed on Treg cells. Interestingly, the way by which LPM from the distal colon sense their microenvironment is completely different from that performed by SI and proximal colon LPM (Table 2). Indeed, distal colon LPM form balloon-like protrusions that insert between colonic epithelial cells but do not extend into the lumen like in the SI (Chikina et al., 2020). They remain instead confined in the intercellular space of the epithelium. These balloon-like protrusions, which are induced by the presence of fungi in the lumen, sample the fluids absorbed by epithelial cells to detect toxins among fungi metabolites. By instructing them to stop absorption, MFs with balloon-like protrusions protect colonic epithelial cells from dying of absorbing too much fungal toxins (Chikina et al., 2020).

## Specificity of Intestinal Immune Inductive Site Macrophages

As mentioned above, the gastrointestinal mucosa of vertebrates has specialized sites dedicated to the detection of pathogens in contaminated food and water. Indeed, in reptiles, amphibians, and lungfishes, the gut contains lymphoid aggregates resembling the ILFs found in mammals (Borysenko and Cooper, 1972; Zapata and Solas, 1979; Solas and Zapata, 1980; Tacchi et al., 2015; Ashford et al., 2019). Like mammal ILFs, the number and size of these aggregates increase with microbial challenges (Tacchi et al., 2015; Ashford et al., 2019). Based on recent observations made on lungfish, it seems however that these lymphoid aggregates lack a well-structured organization, showing no segregation between B and T cells, no germinal center, no AID expression, and no somatic hypermutation (Tacchi et al., 2015). The cellular composition of these primitive aggregates is otherwise poorly described; and MFs, although observed by electron microscopy (Ardavín et al., 1982), have not been well-characterized.

In addition to ILFs, mammals and also birds have PPs that are distributed along the SI, especially in the last part of the ileum (Jung et al., 2010). PPs consist of clustered B cell follicles forming domes on the surface of the mucosa. These domes are separated from each other by dome-associated villi (DAV) over interfollicular regions (IFRs) enriched in T cells. A specialized follicle-associated epithelium (FAE) separates the subepithelial dome (SED) above the follicle from the gut lumen. This FAE provides a permissive environment for pathogen entry. Indeed, it secretes no or few IgA and antimicrobial proteins and is covered by a reduced mucus layer. This is due to lack of polymeric Ig receptor expression, inhibition of IL-22 signaling, and diminished number of goblet cells (Bhalla and Owen, 1982; Pappo and Owen, 1988; Jinnohara et al., 2017). Moreover, the glycocalyx is attenuated over the FAE favoring interaction of luminal antigens with the mucosal surface (Frey et al., 1996; Mantis et al., 2000). Finally, the specialized FAE cell termed M cell efficiently binds and transports all kind of antigens from the lumen to the SED (Ohno, 2016; Kobayashi et al., 2019). Therefore, PP MFs are continually exposed to much more threatening elements than other intestinal MFs. They are accordingly equipped with a whole arsenal against pathogens and prone to promote an inflammatory response (Bonnardel et al., 2015; Wagner et al., 2018). Until now, these MFs have been mainly characterized in mice and to a much lesser extent in humans (Table 2). At the exception of DAV MFs that closely resemble LPM of standard villi, other PP MFs are profoundly different from all other MF populations (Wagner et al., 2018). This is exemplified by their lack of F4/80 and CD64 expression in mice and of CD163 in humans (Bonnardel et al., 2015; Wagner et al., 2018, 2020). Nevertheless, they share with most, if not all, mouse MFs the expression of the apoptotic receptor MerTK and high levels of the chemokine receptor CX_3_CR1, both markers enabling their distinction from conventional DC (Bonnardel et al., 2015, 2017; Wagner et al., 2020). In relation to their important role in innate defense, PP MFs express very large amounts of the antibacterial protein lysozyme, which was the first reliable marker to identify monocyte-derived cells in PPs of mice, rats, and humans (Lelouard et al., 2010). This has given rise to their LysoMac nickname for lysozyme-expressing MFs (Bonnardel et al., 2015). Interestingly, monocytes give also rise in PPs to the unique lysozyme-expressing DC termed LysoDC. LysoDC have a transcriptional program close but not identical to that of PP MFs as they display additional functions, notably in terms of antigen presentation (Bonnardel et al., 2015; Martinez-Lopez et al., 2019; Wagner et al., 2020). Like conventional DC, mature LysoDC are indeed able to prime naïve T cells at least *in vitro* for IFNγ and IL-17 production (Bonnardel et al., 2015; Martinez-Lopez et al., 2019). This ability is strengthened by stimulation with a TLR7 ligand. In addition, TLR7 stimulation induces expression of CCR7 by subepithelial LysoDC and promotes their migration to the periphery of the IFR where they encounter naïve T cells and where they interact tightly with newly activated proliferative T cells (Wagner et al., 2020). At steady state, very few if any LysoDC are in the IFR, and only few of them are located in the follicle, with most of them being in the SED where they excel in antigen capture. Conversely, MFs have been observed in all regions of PPs (Bonnardel et al., 2015). In addition to LysoMac, mainly located in the SED, the follicle, and the IFR, there are indeed muscularis and serosal MFs, and germinal center tingible body MFs (TBM). Interestingly, these different anatomic locations are tightly linked to phenotypic distinctions between PP MFs (Table 2). Thus, muscularis and serosal MFs below the IFR express CD169, whereas other PP MFs do not. As well, TBM and interfollicular and lower follicular LysoMac express the phosphatidylserine receptor TIM-4, whereas subepithelial and upper follicular LysoMac do not. This suggests that an important regional specialization of MF functions exists inside the PPs itself (Wagner et al., 2018). Thus, TIM-4 mainly expressed by MFs in the regions of T cell priming and B cell selection belongs to the family of apoptotic cell receptors and is known to be involved in the regulation of the adaptive immune response and prevention of autoimmunity through removal of both B and T cells (Albacker et al., 2010, 2013; Rodriguez-Manzanet et al., 2010). Therefore, TIM-4^+^ MFs could protect PPs from an exaggerated inflammatory reaction by regulating both T and B cell numbers.

As mentioned above, TIM-4^−^ LysoMac as well as LysoDC are close to the FAE, and they play key role in the uptake of particulate antigens and pathogenic bacteria (Lelouard et al., 2010, 2012; Disson et al., 2018). The mechanisms by which LysoDC and TIM-4^−^ LysoMac sample luminal antigens are different from either SI or colonic LPM. Indeed, phagocytosis of antigens by LysoDC and TIM-4^−^ LysoMac either follows M cell transcytosis or occurs through LysoDC dendrite extension into the lumen through M cell-specific transcellular pores (Lelouard et al., 2012; Bonnardel et al., 2015). Therefore, M cells tightly control both mechanisms. Accordingly, absence of M cells is associated with a strong downregulation of antigen uptake in PPs and of IgA production in villi (Rios et al., 2016). Unlike villus paracellular transepithelial dendrites, these LysoDC trans-M cell dendrites do not depend on CX_3_CR1 expression (Bonnardel et al., 2015). LysoDC and TIM-4^−^ LysoMac also influence FAE properties to favor contact with exogenous antigens. Thus, they express *Il22ra2*, which encodes IL-22BP, an inhibitor of Il-22 (Da Silva et al., 2017). IL-22BP promotes microbial sampling by influencing the FAE transcriptional program, notably by inhibiting genes encoding antimicrobial proteins and also genes involved in surface glycosylation and mucus production (Jinnohara et al., 2017). PP MFs are also likely involved in M cell differentiation as long-term blockade of CSF1R, which is known to deplete MFs, impairs M cell differentiation (Sehgal et al., 2018). Finally, together with other immune cells, they could be involved in M cell maturation via the expression of the S100 family member S100a4 (Kunimura et al., 2019).

More globally, in relation to the fact that PPs are a permissive entry site for a large number of pathogens, PP MFs display a strong antiviral and antibacterial transcriptional program (Bonnardel et al., 2015). Moreover, PP MFs lack the typical anti-inflammatory properties of other intestinal MFs (Wagner et al., 2018). Thus, they do not produce IL-10 or express its receptor but instead secrete TNF and IL-6 upon stimulation (Bonnardel et al., 2015; Wagner et al., 2018). Therefore, unlike most intestinal MFs, they retain a strong ability to promote inflammation.

## Conclusions and Perspectives

There has been incredible progress in recent years in the appreciation of intestinal MF heterogeneity (Table 2). This obviously raises great hope for targeted therapies that would render possible to alter a defective population without disturbing the others or to promote one population over the others and thus restore homeostasis. However, much more work is needed to understand the signaling between MFs and their direct neighbors and how this can be used to remodel MF properties. As suggested by Guilliams et al. (2020), manipulation of the neighboring cells that imprint the MF with its functional properties instead of the MF itself could represent alternative and interesting strategies for deciphering the mechanisms that dictate the fate of intestinal MFs on the one hand and modifying key instructing factors according to pathologies on the other hand. Nutritional- and microbial-based intervention strategies to modulate intestinal MF properties have also become a promising therapeutic approach to treat and prevent intestinal diseases. A great challenge for all these approaches will be to deal with the complexity of the structure and diversity of potentially simultaneous signals (food, microbiota, pathogen, stromal, and immune cell-derived factors) that make the intestine such a special and diversified organ.

## Author Contributions

CAP, JT, and HL: writing—original draft. CAP, JT, HL, and J-PG: writing—revision. CAP: figure design. HL and J-PG: funding acquisition. All authors contributed to the article and approved the submitted version.

## Conflict of Interest

The authors declare that the research was conducted in the absence of any commercial or financial relationships that could be construed as a potential conflict of interest.
